# Activity of daily living and its associated factors in war survivors with no visual acuity

**Published:** 2010

**Authors:** Reza Amini, Hamid Haghani, Mehdi Masoomi, Shervin Assari

**Affiliations:** a Janbazan Medical and Engineering Research Center, Tehran, Iran; b Statistics Department, School of Management and Medical Information & Health Sciences, Iran University of Medical Sciences, Tehran, Iran

**Keywords:** War, No Visual Acuity, Activity of Daily Living

## Abstract

**BACKGROUND:**

War is a known cause of tremendous physical injuries to different body organs, and eyes are not exceptions. War-related no visual acuity (NVA) affects both the victim and the family. Activity of daily living (ADL) can display personal life independency and is considered as a morbidity index. This study was designed to investigate the ADL profile of war survivors with NVA.

**METHODS:**

This cross-sectional study was conducted in 2007 in Iran. In this study, 500 Iranian people with war related NVA were invited to take part in a camp in Mashhad city. ADL was evaluated using Barthel Index and demographic data were collected using a data sheet. Stepwise linear regression was used to determine the associates of ADL.

**RESULTS:**

The overall response rate to the invitation was 50%. From the total 250 participants 96.5% were male with a mean age of 43 ± 8 years. Only 8.3% had no dependency in ADL and other 91.7% had some ranges of dependency in at least one of the daily living activities. ADL score was higher in highly educated participants, those younger than 50 years old, those with less co-morbid physical problems (hearing loss) and those with regular physical exercises. According to regression analysis, age and duration of war related NVA were significant predictors of ADL.

**CONCLUSIONS:**

According to the results, both age and the time passed from war related NVA increase the dependency of people with war related NVA.

Different physical injuries - affecting human health - can occur as consequence of war. Loss of visual acuity is by far one of these unwanted consequences of war. Bullets, bursts, burning and chemical agents all can cause severe visual impairment in people participating in wars.^1^

Severe visual impairment can heavily affect people and their family. It decreases social participation.^2^–^5^ To find out dependency consequences of NVA, some studies has been designed to assess the effects of NVA on ADL,^6^–^10^ which are mostly surveys.

Different measures are used in assessment of being handicap followed by NVA.^4^,^11^,^12^ ADL can be used as a screen test that evaluates some aspects of personal daily life such as clothing and bathing. It can show how much somebody is independent in daily activities. ADL studies can determine people who need more special services to maintain personal independency.^13^,^14^ High dependence for ADL can be even a predictor for death.^14^,^15^

This study was designed to investigate the ADL profile of people with war related NVA.

## Methods

### 

#### Design and Setting

This is a cross sectional study on Iranian people with war related NVA in 2007. Five hundred people with war related NVA were invited by Martyrs Foundation through provincial offices to take part in a camp in Mashhad city.

#### Participants

Participants included 250 out of 500 survivors with NVA who accepted the invitation and came from all the provinces. There was no special exclusion criterion. The total number of people with war related NVA in Iran is 700.

#### Data Collection

For the assessment of ADL, we used Barthel index, which is one of the most famous instruments for ADL study. Barthel index evaluates 10 aspects of daily life including feeding (0 = unable, 5 = needs help cutting, spreading butter, etc., or requires modified diet, 10 = independent), bathing (0 = dependent, 5 = independent [or in shower]), grooming (0 = needs to help with personal care, 5 = independent face/hair/teeth/shaving [implements provided]), dressing (0 = dependent, 5 = needs help but can do about half unaided, 10 = independent (including buttons, zips, laces, etc.), bowels (0 = incontinent [or needs to be given enemas], 5 = occasional accident, 10 = continent), bladder (0 = incontinent, or catheterized and unable to manage alone, 5 = occasional accident, 10 = continent), toilet use (0 = dependent, 5 = needs some help, but can do something alone, 10 = independent [on and off, dressing, wiping]), transfers (bed to chair and back) (0 = unable, no sitting balance, 5 = major help (one or two people, physical), can sit, 10 = minor help (verbal or physical), 15 = independent), mobility (on level surfaces) (0 = immobile or < 50 yards, 5 = wheelchair independent, including corners, > 50 yards, 10 = walks with help of one person [verbal or physical] > 50 yards, 15 = independent [but may use any aid; for example a stick] > 50 yards), and stairs (0 = unable, 5 = needs help [verbal, physical, carrying aid], 10 = independent). Total score ranges between 0 and 100.^16^

Demographic data were collected through a data sheet. Data gathering team was trained just before the process in the study field. The mean time for each part of the assessment was about 15 minutes. Every data sheet and questionnaire was checked on the site and data cleaning was done in the field of the study.

#### Statistical Analysis

Statistical analysis including stepwise regression, independent sample t test, ANOVA, Chi square and Tukey were done by a professional statistician.

## Results

### 

#### Participants

Out of 500 people invited for the event, 250 (50%) agreed to participate. The mean age was 43 ± 8 years. The period of NVA ranged from 1 to 29 years, but 79.2% were afflicted to NVA for more than 20 years.

Among the participants, 96.5% were male, 80.9% were jobless, 94.4% were married, and 31.1% had an undergraduate degree. The main frequent cause of NVA was explosion (98%). Only 18.7% reported their NVA as their single impairments, and others had at least one other co-morbidity. The most common injury was burst hit to face. (Table 1)

**Table 1:** Socio-demographic data of the participants (n = 250)

Characteristic	n	%
**Socio-demographic data**		
**Gender:**		
Male	248	96.5
Female	2	3.5
**Economic status:**		
Jobless	202	80.9
Financially under martyr foundation support	48	76.3
**Marital status:**		
Married	236	94.4
Single	14	5.6
**Educational level:**		
Under graduated	78	31.1
High school diploma	71	28.3
Associate degree	9	3.6
Bachelor degree	52	20.7
Master degree	33	13.5
Doctoral degree	7	2.8
**Clinical data**		
**Co-morbidity:**		
Absent	47	18.7
Present	203	81.3
**Number of co-morbid conditions:**		
1	75	30.0
2	53	21.0
3	29	11.7
4	29	11.7
5	17	6.9
**Cause of no visual acuity:**		
War explosion	245	98.0
Other causes	5	2.0

#### Associated Factors of ADL

Independency in ADL was reported by 8.3%, while 91.7% were dependent at least in one daily life activity. Highly educated participants had higher ADL scores (p = 0.012). Number of injuries was associated with ADL (p = 0.034). Age of 50 years was a marginal level for ADL independency decline. Co-morbid injuries significantly declined ADL score.

#### ADL Regressors

According to the regression analysis, age and duration of NVA were significant predictors of ADL. (Table 2)

**Table 2:** Regressors of activity of daily living in Iranian war related blindness (n = 250)

Characteristic	OR	P	95% Confidence Interval for OR	
			Lower Bound	Upper Bound
Age	-0.420	< 0.001	-0.591	-0.249
Duration of no visual acuity	0.619	< 0.001	0.331	0.907

## Discussion

According to the results of this study, age and the time passed since NVA both increase the dependency of people with war related NVA.

The mean age of people in the world has increased within the second half of the 20^th^ century due to decrease of birth rate and increase of life span, so that the population of people over the age of 65 during 2010 to 2030 would be significantly higher than previous decades. As the process of ageing continues, most of the fatal factors will change to disability factors. Ageing accompanied by disability makes the situation worst and requires more rehabilitation services to live independently especially in ADL.^17^–^19^

According to the literature, ADL declines following NVA, and some believe that there is no relation between the cause of NVA and lack of health related quality of life (HRQOL) and ADL independency.^20^,^21^

NVA is a cause of not only morbidity (as presented here), but also mortality. NVA increases the risk of death by 1.4 times, apart from any complication and dependency occurring after NVA. If the functional problems are included in this model, the risk of death for this group will increase 38.1% more than general population. Therefore, prevention is more cost-efficient than service providing, although rehabilitation services can be effective and helpful in current situations.^22^

There is no accurate information about the burden of NVA in developing countries, and nor in Europe.^23^–^26^ The current study as a cross-sectional one can provide a baseline for further studies to plan for the times when there is no reliable or accurate information.^27^

Similar to other types of NVA, war related NVA is a costly phenomenon. Rehabilitation and care services for one NVA patient may cost annually between 9749 to 26720 dollars.^28^ Cost of all NVA (not only the war related) has been estimated about 4.4 million dollars in India,^29^ and 4 billion dollars in the United States.^30^

Some studies presented other classifications. For example, if there were some problems in mobility, bladder control, bowel and bathing, there would be major difficulties in ADL.^13^ A study on Mexican citizens in the United States used the data of 2800 records of an epidemiology assessment of old age and found a significant correlation between ageing and ADL decrease. However, some other factors such as education level, high blood pressure, diabetes mellitus, low self-reported health status, and admission had significantly effects on ADL. Therefore, at least 50% of cases needed help in at least one of the daily activities. But the visually impaired and visually intact people had less dependency and needed less help.^31^–^32^

According to the results of the current study, 91.3% of the survivors with NVA had some dependency. Ageing decreases ADL. In a study in the United States on people with mean age of 72 years, 32% were dependent for bathing, 37% for clothing, 11% for feeding and toilet use, 10% for bed to chair transfer, 55% for transfer to stairs and 13% for mobility on ground level surfaces. A study conducted by Brench and Horwitz compared two groups of with and without visual impairment old people (over 66 year). They found that visual impairment can reduce ADL and lead to more dependency as compared with those without visual problem, especially in physical and emotional daily living.^31^,^33^

According to this study, the war survivors with NVA face many serious problems in ADL and these problems increase with ageing due to some physical impairments and disabilities.

A study that compared ADL in two groups of physically impaired people with and without visual problems found that visually impaired people had significantly lower ADL scores and a periodic rehabilitation was suggested for them. It shows that rehabilitation is really important to NVA people.^22^,^31^ Another study was conducted in a daily clinic on the elderly people with visual impairment and hearing loss to assess their ADL and personal independency. The study had two historical and prospective parts and was conducted in a period of 6 years from 1986 to 1992. According to the results of this study, people with no visual problem had a better score of ADL comparing with visually impaired ones. The result of hearing loss was also the same as visual impairment. Therefore, ADL in visually impaired people and those who had hearing loss was significantly lower than normal population.^15^ Hearing loss has an important role in compensation with NVA especially in acute phase.^31^–^35^ Old age American veterans (over 65 years old) were dependent in at least one of daily activities.^36^ Systematic and organized health and rehabilitation services reduced their age related dependency.^37^

Nowadays, most of the rehabilitation programs aim to improve functional status.^14^ Even though, the effectiveness of rehabilitation in people with NVA is less than those with normal visual acuity, it greatly and deeply changes their level of independency,^38^,^39^ and they will have more independency especially in daily living and participation.

## Conclusions

To conclude, age and duration of NVA can deteriorate the dependency of people with war related NVA. The researchers hope that this study can be a baseline for ADL studies on war survivors with NVA. The results can yield intervention studies and increase their independency.
